# Black Goji Berry (*Lycium ruthenicum* Murray): A Review of Its Pharmacological Activity

**DOI:** 10.3390/nu15194181

**Published:** 2023-09-27

**Authors:** Ho Seon Lee, Chang-Ik Choi

**Affiliations:** Integrated Research Institute for Drug Development, College of Pharmacy, Dongguk University-Seoul, Goyang 10326, Republic of Korea; ghtjsrhtn@naver.com

**Keywords:** black goji berry, black wolfberry, *Lycium ruthenicum* murray, pharmacological activity

## Abstract

*Lycium ruthenicum* Murray (LRM; commonly known as black goji berry or black wolfberry), a plant in the Solanaceae family, grows in the deserts of China’s Qinghai–Tibet plateau. LRM is widely consumed in traditional Chinese medicine, and its fruits are frequently used as herbal remedies to treat heart disease, fatigue, inflammation, and other conditions. Many studies have reported that LRM is rich in functional phytochemicals, such as anthocyanins and polysaccharides, and has various pharmacological actions. This article reviews research on the biological and pharmacological effects of the constituents of LRM fruits. LRM has various pharmacological properties, such as antioxidant, anti-inflammatory, anti-radiation, immune-enhancing, anti-tumor, and protective effects. LRM has much promise as a dietary supplement for preventing many types of chronic metabolic disease.

## 1. Introduction

For thousands of years, medicinal plants have been recognized as beneficial to human nutrition and health, and berries such as blueberry (*Vaccinium* spp.), raspberry (*Rubus idaeus* Linnaeus), cranberry (*Vaccinium oxycoccos* L.), bilberry (*Vaccinium myrtillus* L.), and goji berries (genus *Lycium*) are examples of such fruits [1]. Berries include numerous vitamins, minerals, and phytochemicals shown in in vivo and in vitro studies to promote health and protect against several chronic diseases [2].

Goji is a plant belonging to the Solanaceae family of the *Lycium* genus, and there are about ninety-seven species and six variants [3]. Besides the three medicinal species (*Lycium barbarum* L., *L. chinense* Miller, and *L. ruthenicum* Murray [LRM]) in China, goji berries have been discovered in America, Africa, Europe, and Asia [4]. Goji berries (wolfberries) are noted for red fruits as *L. barbarum* L. and *L chinense* Mill., which are mainly used in Chinese soups, herbal teas, tinctures, wines, and juices [5,6,7]; LRM, which is also used for medicinal purposes, is known as black goji berry (black wolfberry) and has a different composition [8]. According to Islam et al. [9], red goji berries have a larger quantity of carotenoids, while black goji berries have a higher content of phenol, tannin, and monomeric anthocyanin, and therefore have stronger antioxidant activity. Further, the fruit of LRM is high in phytochemicals such as flavonoids, anthocyanins, and polysaccharides, with antioxidant, anti-inflammatory, anti-radiation, immune-boosting, and anti-tumor properties [10]. These studies can contribute to the commercial food industry by providing an understanding of black goji berries.

We collected studies published in English primarily from PubMed, with secondary use of Google Scholar and Web of Science. To collect studies related to the origin, pharmacological activity, and uses of black goji berries, we used a combination of the following search terms: “black goji berries”, “black wolfberry”, “*Lycium ruthenicum*”, “pharmacological activity”, “antioxidant”, “anthocyanin”, and “polysaccharide”. We thoroughly evaluated and reviewed each paper’s research design, results, and comprehension.

## 2. Phytochemical Composition

**Figure 1:**
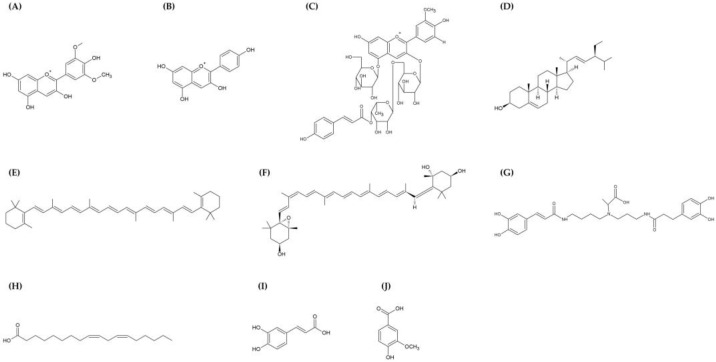
Chemical structure of the main bioactive components in LRM. Anthocyanins: malvidin (**A**), pelargonidin (**B**), peonidin 3-*O*-[6-*O*-(4-*O*-E-*p*-coumaroyl-*O*-α-rhamnopyranosyl)-β-glucopyranoside]-5-*O*-β-glucopyranoside (**C**); phytosterol: stigmasterol (**D**); carotenoids: β-carotene (**E**), neoxanthin (**F**); alkaloid: lyrium spermidine A (**G**); fatty acid: linoleic acid (**H**); phenolic acids: caffeic acid (**I**), vanillic acid (**J**).

A summary of the nutritional components of LRM is shown in Table 1. Carbohydrates were the most prevalent nutrient in LRM, followed by dietary fiber, and the remaining nutrients comprised protein, ash, and fat [11]. LRM is composed mostly of anthocyanins, proanthocyanidins, and carotenoids, as well as fatty acids, essential oils, coumarin and cinnamate derivatives, polysaccharides, alkaloids, and phenolic acids [12]. The majority of LRM research has concentrated on anthocyanins and polysaccharides, and Figure 1 shows the chemical structures of typical LRM components.

## 3. Pharmacological Properties

A summary of the pharmacological activities of LRM is described in Table 2.

### 3.1. Anti-Inflammatory Effects

Inflammation is a marker of numerous diseases and is associated with infection and the immune system, accompanied by the production of inflammatory mediators, the pro-inflammatory cytokines such as tumor necrosis factor-α (TNF-α), interleukin (IL)-1β, and IL-6 [35]. Anti-inflammatory effects have been reported for LRM [13,14,15]. Lu et al. [16] evaluated the regulation of the expression of inflammation-related genes in response to LRM fruit extract treatment. Five-week-old male *ApoE*^–/ –^ mice were randomly assigned into three groups with 10 mice per group. Each group was divided into a normal control (NC) group, Western diet (WD) group, and LRM extract-treated group. The NC group was fed a normal diet, and the other two groups were fed WD for 12 weeks. After administering WD for 8 weeks, 140 mg/kg of LRM extract or distilled water was additionally administered through oral gavages. When the LRM fruit extract-treated group was compared with the NC and WD groups, the expression of the pro-inflammatory gene *Tnf-α* was reduced, but *Il-6* was not significantly changed. In contrast, the expression of the anti-inflammatory gene *Il-4* was increased, while *Il-10* maintained a similar level compared to the WD group. Thus, LRM supplementation reduced inflammation by controlling the expression of *Tnf-α* and *Il-4*.

The nuclear factor (NF)-κB pathway is a response to the pro-inflammatory cytokines TNF-α and IL-1, and plays an important role in the pathogenesis of chronic inflammatory diseases [36]. Chen et al. [17] reported that high and moderate doses of anthocyanin isolated from LRM fruits inhibited d-galactose (d-gal)-induced NF-κB activation and reduced the levels of inflammatory mediators such as cyclo-oxygenase-2, IL-1β, and TNF-α. Anthocyanins isolated from dried LRM fruits were extracted with ethanol.

### 3.2. Anti-Aging Effects

Aging, a growing global problem that has recently received much attention, meets the criteria of the International Classification of Diseases (ICD) [37]. Aging is a complex, time-dependent biological process accompanied by immunologic senescence, altered nutritional sensing, oxidative damage, mitochondrial failure, and other factors [38]. The composition of black goji berries alleviates these factors and contributes to anti-aging [13,14]. Xiong et al. [18] reported that a high dose of LRM fruit extract (10 mg/mL) significantly extended the average lifespan of *C. elegans* by 25.2%. In addition, in a thermal stress assay, the high-dose LRM extract significantly reduced the mortality rate of *C. elegans* by 34.0% compared to the control group (56.9%) (*p* < 0.05). The LRM fruit extract ensured high autonomous locomotivity in *C. elegans*. The proportion of motion A (with great mobility that can move independently and leave sinusoidal trails) nematodes in the control group decreased from 74% to 48% until the mid- to late stages of life (the 15th day), whereas it stayed above 90% in the group receiving large doses of LRM fruit extract. Meanwhile, the LRM fruit extract-treated group had a lower food intake than the control group, and the authors speculated that the goji berry extract could intervene in the calorie restriction pathway for life expectancy extension. Compared to the control group, the LRM-treated group alleviated the aging pigment accumulation, and the high-dose goji berry extract upregulated genes related to lifespan regulation, such as *daf-16*, *sod-2*, *sod-3*, *hsp-16.2*, *sir-2.1*, *daf-12*, and *jnk-1*. Mutations mu86 I, ok434 IV, and sy441 I in the daf-16, sir-2.1, and hsf-1 genes, respectively, reversed the lifespan extension provided by LRM fruit extract, confirming that these genes are required for goji berry-mediated increased longevity.

### 3.3. Anticancer Effects

The primary treatments for cancer include surgery, radiation therapy, and chemotherapy. However, these treatments are often associated with serious side effects, and a key challenge now is to find natural compounds with effective anticancer properties [39]. Here are some research reports on the anticancer properties of LRM [19,20]. Zhang et al. [21] extracted dried LRM fruits by water and ethanol precipitation to obtain crude polysaccharides. LRM polysaccharide 3S-1 (LRP3-S1) was then obtained by purifying it using anion exchange chromatography. Zhang et al. [21] evaluated the antiproliferative activity of the polysaccharide LRP3-S1 by 3-[4,5-dimethylthiazol-2-yl]-2,5 diphenyl tetrazolium bromide (MTT) assay using three pancreatic cancer cell lines: AsPC-1, BxPC-3, and PANC-1. Under treatment with LRP3-S1 for 72 h, proliferation decreased in a concentration-dependent manner, with values of 30.1%, 67.1%, and 29.0%, respectively, for each cell line. Conversely, LRP3-S1 showed an inhibitory ratio of about 10% in HPDE6-C7 and LO2 cells, indicating that LRP3-S1 may not show significant cytotoxicity in normal cell lines. In addition, a cell invasion assay showed that the cellular area in the lower chamber for BxPC-3 cells treated with LRP3-S1 was 92% and 57% at concentrations of 4.36 μM and 8.71 μM, respectively. This indicates that the invasive ability of BxPC-3 cells was attenuated by LRP3-S1 in a concentration-dependent manner. Moreover, p-AKT, p-GSK-3β, p-FAK, and p-p38 were downregulated in a concentration-gradient manner in BxPC-3 cells, suggesting that the FAK/AKT/GSK-3β signaling pathway and the p38 mitogen-activated protein kinase signaling pathway are involved in the regulation of cancer cell growth and invasion by LRP3-S1. Therefore, LRP3-S1-induced downregulation of these associated phosphorylated proteins could decrease pancreatic cancer cell proliferation and invasion ability.

Qin et al. [22] investigated synergistic anticarcinogenic effects and cell-cycle blockade in G0–G1 phase apoptosis (programmed cell death) via a reactive oxygen species (ROS)-dependent pathway under LRM-derived compound treatment. LRM crude polysaccharide was obtained by water extraction followed by ethanol precipitation, and LRM polysaccharide 4 (LRP4) was purified using an ion exchange column and gel permeation column. LRM anthocyanins were obtained through ethanol extraction. LRP4 and anthocyanins were concentrated by freeze-drying. The synergistic effect of LRP4 and anthocyanins (LRP4&AC), a mixture of 20 µg/mL LRM anthocyanin and various concentrations of LRP4 (150, 300, and 500 μg/mL), was measured. Based on MTT assay, LoVo cells (human colorectal carcinoma cells) were treated with LRP4&AC at concentrations of 150, 300, and 500 μg/mL for 24 h. This treatment reduced the viability of cancer cells to 87.2%, 83.5%, and 72.7%, respectively, in a concentration-dependent manner, and similar results were obtained after 48 h. In addition, lactate dehydrogenase (LDH) increased in a concentration-gradient manner, confirming that LRP4&AC has a cytotoxic effect by inducing cell-membrane lysis in tumor cells. The cytotoxicity of LRP4&AC was not specific to one type of carcinoma cells, since HepG2 cells (human hepatoma cells) displayed stunted development. In contrast, normal cells, RAW 264.7 cells (mouse macrophage cells), showed no significant reduction in cell viability. The antiproliferative effect of LRP4&AC on tumor cells consisted in cell-cycle arrest and induction of apoptosis. LRP4&AC inhibited the replication of LoVo cells by G0–G1 arrest. Cells at the S phase were reduced to 24.1% (150 μg/mL) and 17.8% (500 μg/mL), whereas cells at the G0–G1 phase were significantly increased to 66.2% and 73.9%, respectively. This suggested that LRP4&AC prevented cell progression at the G0–G1 phase, potentially by interfering with the initiation of DNA and histone synthesis. LRP4&AC induced tumor-cell apoptosis. The low dose (150 μg/mL) showed 18.2% of the early apoptotic stage and 16.5% of the late apoptotic stage, but the high dose increased these values to 20.9% and 22.4%, respectively. Additionally, LRP4&AC increased ROS levels in cancer cells in a dose-dependent manner. The low dose increased the ROS level by 19.9% and the high dose (500 μg/mL) by 24.2%. The redox balance of LoVo cells could be disturbed by increased oxidative stress due to ROS generated by LRPS&AC, which could cause apoptosis. The mechanism underlying the anticancer effects of LRP4&AC could involve the interaction of the PI3K/Akt and JAK2/STAT3 pathways, leading to an elevated Bax/Bcl-2 ratio, increased caspase-3 levels, and promotion of mitochondrial-mediated apoptosis.

### 3.4. Protective Effects

#### 3.4.1. Hepatoprotective Effects

Chen et al. [13] showed the alleviating effect of LRM anthocyanin supplementation on d-gal-induced liver damage. LRM anthocyanins were obtained through ethanol extraction from dried LRM fruits, followed by concentration, purification, and freeze-drying. On histologic evaluation, the d-gal-treated group showed an increased unclear structure of hepatocytes and cell necrosis, but this visual evidence was improved by LRM anthocyanin treatment. In addition, intake of LRM anthocyanins reversed the results by reducing d-gal-induced serum AST and alanine transaminase (ALT) levels, and blood LDH concentrations. AST and ALT levels reflect the extent of liver damage, while LDH concentrations reflect the extent of cellular damage and inflammation. Moreover, Fas/FasL contributed to cell death receptor signaling, and LRM anthocyanin treatment alleviated d-gal-induced hepatocyte death by downregulating this mRNA level.

Lu et al. [16] investigated the beneficial impact of LRM fruit extract on the development of cholesterol-enriched, high-fat diet-induced nonalcoholic fatty liver disease (NAFLD). Serum aspartate transaminase (AST) levels were significantly increased in the WD group compared to the NC group, but levels were dramatically reduced in the WD group supplemented with LRM fruit extract. These results suggest that LRM fruit extract supplementation protects against liver injury. Additionally, although severe hepatic steatosis was evident in hematoxylin and eosin (H&E) and oil red O staining results for the WD group, LRM fruit extract significantly reduced the size of hepatic fat droplets in the WD group. Thus, LRM fruit extract supplementation had a protective function in cholesterol-induced NAFLD.

#### 3.4.2. Neuroprotective Effects

A slow progressive loss of neurons leads to the progression of neurodegenerative diseases such as Alzheimer’s disease, Parkinson’s disease, amyotrophic lateral sclerosis, multiple sclerosis, Huntington’s disease, and multiple system atrophy [40]. Brain-related aging, and the resulting oxidative stress, inflammatory responses, and apoptosis, are the most important causes of neurodegenerative diseases [41]. There are some reports of neuroprotective activity for black goji berries [14,23].

Chen et al. [17] reported a mechanism for LRM anthocyanin’s potential neuroprotective effect in d-gal-treated rats. LRM anthocyanins were obtained through ethanol extraction from dried LRM fruits, followed by concentration and purification. Female Sprague Dawley rats (12 weeks old, average weight 200–40 g) were divided into five groups of 11 rats each: a control group given normal saline; a d-gal group (100 mg/kg); and three LRM anthocyanin groups by concentration (100 mg/kg of d-gal + 50, 100, or 200 mg/kg of LRM anthocyanins). In the Morris water maze test, the d-gal group had impaired spatial learning and memory, but the LRM anthocyanin groups recovered from these memory impairments. On a day-5 swimming-path test, the control group found the best platform, but the d-gal group found a platform without a specific direction, and the LRM anthocyanins groups performed better than the d-gal group in platform finding. Notably, performance in the high-dose LRM anthocyanin group was comparable to that in the control group. The probe test was performed on day 6, and high- and medium-dose LRM anthocyanins significantly reduced latency to the platform in the d-gal group. Compared to the d-gal group, the LRM anthocyanin groups had a dramatically enhanced number of crossings and time spent in the target quadrant, indicating that LRM anthocyanins may lessen the memory impairment caused by d-gal. In step-down-type passive avoidance tests, the number of errors in the d-gal group was significantly greater than in the control group; medium- (*p* < 0.05) and high-dose (*p* < 0.01) LRM anthocyanins significantly reduced the number of errors. The d-gal group also showed a significant reduction in step-down latency (*p* < 0.001), but medium- and high-dose LRM anthocyanins markedly prolonged step-down latency (*p* < 0.01). These findings suggest that LRM anthocyanins improved memory function and passive avoidance behavior in the d-gal group. The d-gal-treated model causes brain senescence and can lead to animals developing symptoms similar to Alzheimer’s disease. Intracorporeal free amines and d-gal can combine to produce advanced glycation end products (AGEs), and AGEs and their receptors (RAGEs) can increase ROS and inflammatory factors, and eventually impair cognitive performance. Western blot results showed that RAGE expression increased significantly in the d-gal versus the control group, whereas RAGE expression was significantly reduced by LRM anthocyanins. Moreover, various brain insults stimulate microglia/astrocytes, which are components of the neurovascular unit, and neuroinflammation is greatly influenced by the interaction between these two cell types [42]. Ionized calcium-binding adaptor molecule-1 and glial fibrillary acidic protein are markers indicating active microglia and astrocytes, respectively. Western blot results showed that these two markers were elevated in the d-gal group but were significantly suppressed by LRM anthocyanins (*p* < 0.05).

**Figure 2:**
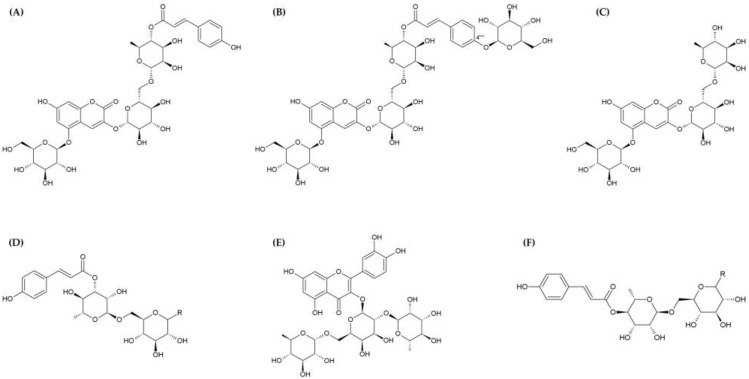
Chemical structure of polyphenolic glycosides isolated from LRM with neuroprotective effects [32]. Compound **1**: lyciumserin A (7-hydroxycoumarin-3-*O*-[6-*O*-(4-*O*-*p*-*trans*-coumaroyl)-α-L-rhamnopyranosyl]-β-D-glucopyranosyl-5-*O*-β-D-glucopyranoside) (**A**), compound **2**: lyciumserin B (7-hydroxycoumarin-3-*O*-[6-*O*-(4-*O*-(4-*O*-β-d-glucopyranosyl)-*trans*-*p*-coumaroyl)-α-L-rhamnopyranosyl]-β-D-glucopyranosyl-5-*O*-β-D-glucopyranoside) (**B**), compound **3**: lyciumserin C (7-hydroxycoumarin-3-*O*-(6-*O*-α-L-rhamnopyranosyl)-β-D-glucopyranosyl-5-*O*-β-D-glucopyranoside) (**C**), compound **5**: lyciumserin E (6-*O*-(3-*O*-*p*-*trans*-coumaroyl-α-L-rhamnopyranosyl)-β-D-glucopyrano-side) (R: β-OH) and compound **6**: lyciumserin F (6-*O*-(3-*O*-*p*-*trans*-coumaroyl-α-L-rhamnopyranosyl)-α-D-glucopyranoside) (R: α-OH) (**D**), compound **11**: puchikrin (**E**), compound **16**: 6-*O*-(4-*O*-*p*-*trans*-coumaroyl-α-L-rhamnopyranosyl)-α-D-glucopyranosyl-methanol (R: α-OCH3), compound **17**: 6-*O*-(4-*O*-*p*-*trans*-coumaroyl-α-L-rhamnopyranosyl)-β-D-glucopyranoside (R: β-OH), and compound **18**: 6-*O*-(4-*O*-*p*-*trans*-coumaroyl-α-L-rhamnopyranosyl)-α-D-glucopyranoside (R: α-OH) (**F**).

Hu et al. [24] evaluated the neuroprotective activity of polyphenolic glycosides compounds **1** (lyciumserin A), **2** (lyciumserin B), **3** (lyciumserin C), **5**/**6** (lyciumserin E and F), **11** (puchikrin), **16** (6-*O*-(4-*O*-*p*-*trans*-coumaroyl-α-L-rhamnopyranosyl)-α-D-glucopyranosyl-methanol), and **17**/**18** (6-*O*-(4-*O*-*p*-*trans*-coumaroyl-α-L-rhamnopyranosyl)-β-D-glucopyranoside and 6-*O*-(4-*O*-*p*-*trans*-coumaroyl-α-L-rhamnopyranosyl)-α-D-glucopyranoside) isolated from LRM extracts. Lyciumserin A, B, C, E, and F are newly named compounds first isolated by Hu et al. [24] Figure 2 shows the chemical structures of these compounds. In PC12 cells, the neurotoxin 6-hydroxydopamine hydrobromide (6-OHDA) reduced cell viability to 51.9% compared to the control group. In contrast, when treated with compounds **1**–**3** and **5**/**6** (50 and 100 μM), cell viability was restored to almost 70%, confirming that the neurotoxic effect of 6-OHDA was delayed. Notably, 100 μM of compound **2** showed the highest neuroprotective effect (72.4%), which was higher than that for rasagiline, a positive control group (70.5%). Based on this protective effect, the changes in PC12 cell morphology were evaluated. Pre-treatment with compounds **1** (50 μM) and **2** (100 μM) considerably improved morphologic alterations compared to cells treated with 6-OHDA alone, which caused evident cell shrinkage and poor adherence. In addition, neuroprotective effects for selected compounds were demonstrated by staining cells with fluorescein diacetate (FDA) and propidium iodide (PI). FDA stains viable cells, whereas PI stains damaged cells. Compounds **1** and **2** increased FDA-stained cells and decreased PI-stained cells. These findings imply that compounds 1 and 2 can prevent 6-OHDA-induced apoptosis in PC12 cells. Meanwhile, compounds **1**, **2**, **11**, **16**, and **17**/**18**, which are some of the phenolic glycosides in LRM, exhibited monoamine oxidase B (MAO-B) inhibition of more than 50% at a concentration of 100 μM. The 50% inhibitory concentration (IC_50_) values for each compound were 60.7 ± 1.7 μM (compound **1**), 22.2 ± 0.8 μM (compound **2**), 79.3 ± 2.4 μM (compound **11**), 68.9 ± 1.5 μM (compound **16**), and 75.5 ± 3.8 μM (compound **17**/**18**). MAO-B inhibitors can reduce neurotoxin formation by preventing dopamine oxidation, and by reducing free-radical formation due to monoamine oxidation. MAO-B inhibitors continue to be developed for the treatment of Parkinson’s disease [43,44]. Notably, compound **2** has both MAO-B inhibitory activity and neuroprotective activity, and the authors reasoned that this is because compound **2** is the only structure with a *p*-coumaric acid moiety, and a glucose moiety located at C-4‴’ (Figure 2B). 

#### 3.4.3. Cardioprotective Effects

Cardioprotection refers to the ability of cardiac muscle to endure injuries such as metabolic stress or ischemia-reperfusion [45]. Yossa Nzeuwa et al. [25] showed that an aqueous extract of dried fruits of black goji berry was effective against isoproterenol-induced acute myocardial ischemia. A total of forty Institute of Cancer Research (ICR) mice were randomly assigned to five groups of eight mice each. Group I (the normal group) and Group II (the negative control group) mice received 1.0 mL of 0.5% saline intragastrically each day. For 8 days, groups III and IV received LRM fruit extract suspension (375 mg/kg or 750 mg/kg), and group V received propranolol (positive control group; 15 mg/kg) through intragastric intubation. The mice in Groups II, III, IV, and V received two isoproterenol (20 mg/kg) intraperitoneal injections repeated 24 h apart on days 7 and 8. Creatine kinase-myocardial band (CK-MB) and LDH are myocardial marker enzymes present in cardiac muscle cells; they escape into the blood when myocardial necrosis occurs [46]. In the isoproterenol-alone group (Group II), the levels of both enzymes were significantly increased (*p* < 0.001) compared to the control group (Group I); however, pretreatment with high-dose LRM fruit extract (750 mg/kg; Group IV) led to significantly reduced levels of both enzymes (*p* < 0.05). These results were consistent with previous studies, in which LRM fruit extract protected against cardiac injury induced by overexertion in rats [47]. In addition, histopathologic examination showed that low-dose (Group III) and high-dose LRM fruit extract (Group IV) were associated with reduced myocardial tissue damage compared to the isoproterenol-alone group (Group II). Thus, in myocardial tissue treated with LRM fruit extract, inflammatory cell infiltration, and traces or signs of cellular edema and necrosis were reduced or returned to normal.

#### 3.4.4. Protection against Radiation Injury

Ionizing radiation particles pose a major threat to human health because of their physical ability to generate free radicals that cause direct and indirect DNA damage [48]. Amifostine (WR-2721) is the only cytoprotective agent approved by the US Food and Drug Administration as prophylaxis to protect against radiation injury; however, widespread use of amifostine is limited due to side effects such as hypotension, vomiting, and nausea [49,50]. Duan et al. [26] evaluated the radioprotective effect of LRM fruit extract. A total of 180 Kunming mice were randomly assigned in groups of 60 according to irradiation (days 3, 7, and 14). Each group was designated as a control group, model group, positive drug group (amifostine 150 mg/kg), or LRM fruit extract-treated group (2, 4, or 8 g/kg). All mice, except those in the control group, were exposed to 5 Gy of X-ray radiation at one time distributed equally across the entire body. On day 3, 7, and 14 following irradiation, measurements of body weight, hemogram, thymus index, spleen index, DNA, caspase-3, caspase-6, and P53 were carried out. LRM fruit extract showed a preventive effect against weight loss in irradiated mice but did not show significant results on the hemogram (leukocytes, erythrocytes, hemoglobin, and thrombocytes). The thymus index increased significantly (*p* < 0.05) in the LRM fruit extract middle- and high-dose groups on day 3 and 7 after irradiation. In addition, the spleen index increased significantly (*p* < 0.05) in the low- and middle-dose groups on day 14, compared to the model group. Further, the DNA content increased significantly (*p* < 0.05) with all LRM fruit extract doses on day 3, and with the low and middle LRM fruit extract doses on day 7 and 14, compared to the model group. Moreover, the low- and middle-dose LRM fruit extract-treated groups showed significantly decreased levels of caspase-6 on day 7 after irradiation (*p* < 0.05), and significantly decreased levels of caspase-3 on day 14 after irradiation (*p* < 0.05), compared to the model group. The caspase results supported the P53 data, and immunohistochemical images showed that P53 expression was decreased in the LRM fruit extract-treated group.

### 3.5. Immunomodulatory Effects

Nitric oxide (NO), a signaling molecule that can modulate immunity, is mainly produced by macrophages [51]. The NO generated changes the phenotype of macrophages from M2 to M1 [52]. While the M1 phenotype suppresses tumors by phagocytosis, the M2 phenotype subdues the activated immune response, helping tumor progression [53]. Lipopolysaccharides (LPS) are agents that stimulate macrophage NO production, but their clinical application is limited due to side effects and dose-limiting toxicity [52]. Peng et al. [27] assessed the immunomodulatory activity by measuring NO production by LRM polysaccharide pectin-5 (LRPP5) in RAW264.7 macrophages. LRPP5 was obtained from crude polysaccharides extracted (ethanol precipitation after water extraction) from LRM fruit powder and purified by ion exchange and gel permeation chromatography. An MTT assay was performed to observe the effect of LRPP5 (50, 100, 200, 500, and 1000 μg/mL) on cell viability in RAW264.7 cells treated for 24 h. Cell viability was dramatically enhanced at LRPP5 concentrations of 500 and 1000 μg/mL; with the highest concentration, cell viability was enhanced approximately 3.1-fold compared to the control group. At lower LRPP5 concentrations, there was no apparent effect. An LRPP5 concentration of ≤200 μg/mL, with no appreciable change in cell viability, was selected for NO estimation. NO produced by macrophages is converted to nitrite ions, which indicate macrophage activation. LRPP5 treatment (25, 50, 100, and 200 μg/mL) for 24 h significantly increased NO production compared to the control group (*p* < 0.01), suggesting that LRPP5 stimulated macrophages to release NO.

In cyclophosphamide (Cy)-induced immunosuppression in mice, LRM polysaccharide 3 (LRP3) was reported to have immunomodulatory benefits [28]. LRM crude polysaccharide was isolated using dried LRM fruit powder through water extraction and ethanol precipitation. Purified LRP3 was obtained from LRM crude polysaccharide using ion exchange and gel permeation chromatography. Female Kunming mice were randomly divided into five groups (10 mice per group): a control group, a Cy group, and LRP3-treated groups (25, 50, and 100 mg/kg of LRP3) that received an intraperitoneal injection with saline or LRP3 once a day for 10 consecutive days. The spleen and thymus indices in the Cy group were significantly lower than those in the control group (*p* < 0.05), clearly indicating a decreased immunologic response. However, both parameters significantly improved in all LRP3-treated groups compared to the Cy group (*p* < 0.05). These results, showing that LRP3 is involved in the recovery of the spleen and thymus indices in mice, agree with previous findings [26]. In addition, an MTT assay was performed to evaluate the effect of LRP3 on the proliferation of spleen cells. Splenocytes from each group were cultured for 24 h and 5.0 µg/mL of lectin (T cell stimulation) and LPS (B cell stimulation) were added. After 72 h, the proliferative effect on stimulated lymphocytes was confirmed. Under the lectin-treated condition, it was confirmed that proliferation in the LRP3-treated group (25, 50, and 100 mg/kg) was significantly increased (*p* < 0.05) by 55%, 64%, and 76%, respectively, compared to the Cy group. Similarly, under the LPS treatment condition, the LRP3-treated group showed significant increases (*p* < 0.05) in spleen-cell proliferation by 65%, 74%, and 87%, respectively, in a gradient manner compared to the Cy group. Further, the reduced index caused by Cy in the evaluation of macrophage phagocytic function was significantly reversed (*p* < 0.05), in a concentration-dependent manner, by LRP3 pretreatment. Moreover, LRP3 pretreatment demonstrated a regulatory humoral immune-enhancing function by dose-dependently improving (*p* < 0.05) the reduced value produced by Cy in the serum hemolysis production test. Meanwhile, pretreatment with LRM in mice with Cy-induced immunosuppression significantly elevated (*p* < 0.05) the levels of IL-2, IL-6, and TNF-α.

Recently, Xu et al. [29] reported that LRM anthocyanins inhibited the hyperproliferation and aggressive invasion of synovial fibroblasts (SF) in patients with rheumatoid arthritis (RA), thus suggesting the potential for LRM anthocyanins in the treatment of RA. LRM anthocyanins were obtained commercially and purified directly. LRM anthocyanins (100, 200, and 400 μg/mL) significantly decreased (*p* < 0.01) cell viability in a concentration-dependent manner at both 24 and 48 h in an MTT assay. Comparable results were found with the conventional chemotherapeutic drug methotrexate (MTX) 400 g/mL. The findings for LRM anthocyanins from the MTT assay were consistent with results from a Cell Counting Kit-8 assay. In a calcein-acetyl methoxy methyl ester/propidium iodide assay, LRM anthocyanins also inhibited SF proliferation similarly to MTX. Notably, immunosuppressive reactions, a major side effect of MTX, were not observed with LRM anthocyanins. Unlike MTX, LRM anthocyanins 400 g/mL had no effect on T cells and monocyte/macrophage development.

### 3.6. Other Effects

#### 3.6.1. Anti-Fatigue Activity

Control of fatigue is crucial because such control can prevent the development of many serious health problems; indeed, fatigue is regarded as an intermediate condition between health and sickness [54]. Bi et al. [15] reported that LRM fruit water extract has anti-fatigue activity. A total of 80 Kunming mice (sex ratio 1:1) were orally administered with distilled water (control group) or LRM fruit extract (0.05, 0.1, 0.2, or 0.5 mg/g water extract) once a day for 28 days. There was no significant difference in body weight between the control and treatment groups; however, swimming duration increased in a dose-dependent manner compared to the control group. Significant results were observed at all concentrations, with the greatest time increase at the highest dose (*p* < 0.05, *p* < 0.01, *p* < 0.001, and *p* < 0.001 for LRM fruit water extract 0.05, 0.1, 0.2, or 0.5 mg/g, respectively). This suggests that the LRM fruit water extract significantly alleviated exercise-induced fatigue. As described above, LRM had an anti-fatigue effect through the reduction of inflammation and oxidative stress, and an increase in SOD was observed along with decreases in LDH, TNF-α, IL-6, IL-1β, and IL-2.

Ni et al. [30] reported the anti-fatigue activity of polysaccharides in black goji berries. Eight-week-old male BALB/c mice were assigned to five groups (6 mice per group): an intact group (control), p.s. group (as a model group; saline treatment with a swimming test), and LRM polysaccharide-treated groups (50, 100, and 200 mg/kg). Saline was administered to the non-LRM groups, and only the intact group was spared the swimming test. The polysaccharide fraction of hot water-extracted LRM or saline was orally administered to mice once daily for 15 days. In the swimming test, immobility times were significantly reduced (*p* < 0.05) for LRM polysaccharide 50 and 200 mg/kg. Immobility times indicate the degree of fatigue, and their decrease means anti-fatigue activity. For serum biochemical parameters measured after the swimming test, the markers reduced by the forced swim test were restored to normal by LRM polysaccharide. Increases in serum blood urea nitrogen (BUN), creatine phosphokinase (CPK), LDH, and malondialdehyde (MDA) were observed with the progress of the swim test but returned to normal with LRM polysaccharide 100 mg/kg and 200 mg/kg. Conversely, glucose, superoxide dismutase (SOD), and glutathione peroxidase (GPx) levels reduced by the swim test were increased again with LRM polysaccharide administration.

#### 3.6.2. Anti-Obesity and Antidiabetic Activity

Obesity is caused by an imbalance between dietary energy intake and expenditure [55]. It is an important health problem worldwide because it is associated with metabolic conditions such as type 2 diabetes, hypertension, dyslipidemia, and cardiovascular disease [56]. Obesity is usually managed with diet, physical activity and pharmaceuticals [57]. However, some pharmaceuticals may have serious side effects; thus, there is an increased need for natural products considered relatively safe [58,59]. There is a report related to the anti-obesity effect of LRM extract [16].

Pancreatic lipase is a digestive enzyme that breaks down fat and improves triglyceride (TG) absorption; therefore, blocking pancreatic lipase can have an anti-obesity effect [60]. Cholesterol esterase (CEase) is also a lipolytic enzyme and is involved in the absorption of dietary cholesterol [61]. Zhao et al. [31] reported that LRM seed oil had inhibitory activity against both pancreatic lipase and CEase. For pancreatic lipase, the inhibitory activity of LRM seed oil increased in a concentration-dependent manner, and the IC_50_ value was reported as 12.4 ± 0.1 mg/mL. In addition, the inhibitory activity of LRM seed oil against CEase was enhanced in a gradient manner, and the IC_50_ value was 2.6 ± 0.1 mg/mL, indicating a very strong effect. The inhibitory pattern of p-nitrophenyl butyrate, a substrate of CEase, was reversible, and non-competitive inhibition was determined using a Lineweaver–Burk double reciprocal plot.

In another study by Zhao et al. [32], bioactive phenylpropanoid derivatives isolated from LRM were investigated for potential antidiabetic activity due to α-glucosidase inhibitory activity. Although most compounds showed weak activity, compound **13** (ethyl *p*-*trans*-coumarate isolated from LRM) was superior and had similar effects to the positive control (acarbose).

#### 3.6.3. Activity against Influenza

Viral infections can lead to global problems and, annually, influenza poses a health risk to millions of people worldwide by producing severe respiratory infections [62]. Kurskaya et al. [33] noted the antiviral activity of an LRM fruit extract against influenza A/Novosibirsk/RII-27192S/2020 (H3N2) virus. In an MTT assay using Madin–Darby canine kidney (MDCK) cells, the 50% cytotoxicity concentration (CC_50_) was more than 125 μg/mL, so a concentration range of 15.6–500 μg/mL was used in the experiment. Anti-influenza activity was considered effective when inhibition of A/H3N2 virus-infected MDCK cells of 50% or more was exhibited. The MTT assay results showed that the LRM fruit extract (15.6–125 μg/mL) enhanced the viability of A/H3N2 virus-infected MDCK cells in a concentration-dependent manner compared to the control group.

#### 3.6.4. Tyrosinase Inhibitory Activity

Tyrosinase inhibitory activity reduces skin pigmentation and leads to skin whitening; however, kojic acid, a conventional inhibitor, is cytotoxic. Therefore, natural products with relatively few side effects are attracting attention as possible therapeutic candidates with a tyrosinase inhibitory function. According to the study by Shen et al. [34], dried LRM fruit powder was dissolved in hydrochloric acid aqueous solution and used as LRM extract. In addition, the LRM extract was eluted with a resin column and purified LRM anthocyanins was obtained through freeze-drying. LRM anthocyanins inhibited tyrosinase monophenolase: purified anthocyanin (IC_50_ 1.5 ± 0.06 mg/mL) was superior to LRM extract (IC_50_ 3.0 ± 0.02 mg/mL) but weaker than kojic acid (IC_50_ 3.0 ± 0.02 µg/mL). LRM anthocyanins bound reversibly and competitively to tyrosinase monophenolase when the mechanism was investigated (enzyme inhibition constant; K_i_ 39.8 ± 1.4 mg/mL). The inhibitory activity against diphenolase was also measured, and purified anthocyanin (3 mg/mL, 42.2 ± 0.77%) had a greater inhibitory power than the LRM extract (3 mg/mL, 30.8 ± 1.0%); the inhibitory activity was reversible and non-competitive (enzyme–substrate complex; K_is_ 2.4 ± 0.10 mg/mL). The IC_50_ for kojic acid was 1.90 ± 0.05 µg/mL.

#### 3.6.5. Antioxidant Effects

Antioxidants play a crucial role in preventing oxidative cell damage, which can result in problems such as Alzheimer’s disease, cancer, cardiovascular disease, and chronic inflammation [63]. Anthocyanins, polyphenols from the flavonoid family, are found in practically all fruits and vegetables and provide these foods with their anti-inflammatory and antioxidant capabilities [64]. There have been numerous reports on the antioxidant activity of LRM over the past few years. The antioxidant properties of LRM have been demonstrated through various methods including DPPH (2,2-diphenyl-1-picrylhydrazyl) radical scavenging assay [14,16,31,32,34,65,66,67,68], ABTS (2,2′-azino-bis(3-ethylbenzothiazoline)-6-sulfonic acid) radical scavenging assay [14,31,34,66], hydroxyl radical scavenging assay [34,65,67], SOD radical scavenging assay [13,15,18,23,25,30,34,65,67], lipid peroxidation assay [13,14,16,17,25,30,69], antioxidant enzyme activity assay [13,14,16,17,18,23,25,30,69], ROS measurement [14,16,17,23,69,70], oxygen radical absorbance capacity assay [19,32], peroxyl radical scavenging capacity assay [19], and cellular antioxidant activity assay [19].

## 4. Conclusions

LRM has various pharmacological properties, including antioxidant, anti-inflammatory, anti-aging, anticancer, immunomodulatory, anti-fatigue, anti-obesity, antidiabetic, antiviral, and tyrosinase inhibitory activity. LRM also has protective effects, such as hepatoprotection, neuroprotection, cardioprotection, and radioprotection. Most of these properties are aligned with the roles of polyphenols, flavonoids, anthocyanins, and functional polysaccharides in LRM, and there are many relevant findings. Black goji berry has the potential to contribute to the development of food additives and functional foods as well as the treatment of diseases based on various pharmacological activities and can also be applied in medicine and cosmetics. Clinical research results have been reported for *L. barbarum* and *L. chinense*, well-known as goji berries, but not for LRM. The results are insufficient to confirm safety and efficacy for LRM in humans because most LRM studies are still carried out at the cellular and animal level; therefore, additional clinical research is required. The results discussed in our review paper demonstrate that LRM has the potential to be used in the treatment of a variety of diseases and could also serve as a steppingstone for future clinical research.

## Figures and Tables

**Table 1:** Nutritional information of *Lycium ruthenicum* Murray (LRM) fruits.

Nutrients	g/100 g DW	Nutrients	mg/100 g DW
Total carbohydrates	67.0 ± 1.2	Vitamins	12.42 ± 1.26
Dietary fiber	12.1 ± 0.1	Macroelements	22.1 ± 1.6
Proteins	11.5 ± 0.3	Microelements	129.2 ± 9.1 *
Ash	6.3 ± 0.1	Carotenoids	1.52 ± 0.01
Fat	3.0 ± 0.1	Anthocyanins	25.1 ± 1.3
Organic acids	4.69 ± 0.13	Polysaccharides	31.3 ± 1.6

* = μg/g DW.

**Table 2:** Summary of pharmacological studies for *Lycium ruthenicum* Murray (LRM).

Pharmacological Activity	Tested Substance	Study Model	Dose/Concentration	Study Result(s)	Ref.
**Anti-inflammation**	Anthocyanin	Sprague Dawley rats	100 mg/kg	Decreased: TNF-α and IL-6 Increased: IL-10	[13]
	Anthocyanin	Neuro-2a cells and Male C57BL/6 mice	10 μM (in vitro) and 50 and 100 mg/kg (in vivo)	(in vitro) Decreased: COX-2, TNF-α, IL-6, IL-1β, and p-NF-κBp65 (in vivo) Decreased: p-NF-κB, TNF-α, IL-1β, and IL-6	[14]
	Fruit extract	Kunming mice	0.05, 0.1, 0.2, and 0.5 mg/g	Decreased: TNF-α, IL-1β, IL-2, and IL-6	[15]
	Fruit extract	Male *ApoE*^−/−^ mice	140 mg/kg	Decreased: *Tnf-α* (compared with NC and WD) Not significantly changed: *Il-6* (compared with NC and WD) and *Il-10* (compared with WD) Increased: *Il-4* (compared with WD)	[16]
	Anthocyanin	Female Sprague Dawley rats	50, 100, and 200 mg/kg	Decreased: NF-κB, IL-1β, COX-2, and TNF-α	[17]
**Anti-aging**	Anthocyanin	Sprague Dawley rats	100 mg/kg	Decreased: serum aging markers (AGEs and MDA) Increased: swimming speed Improved: amino acid metabolic disturbance	[13]
	Anthocyanin	Male C57BL/6 mice	50 and 100 mg/kg	Improved: cognitive impairment (enhanced spatial learning and memory abilities)	[14]
	Fruit extract	*C. elegans*	2, 5, and 10 mg/mL	Decreased: mortality rate (for heat shock), motility, lipofuscin (age pigment), reproductive ability, and age-related gene expression (*age-1*) Increased: average lifespan, SOD, CAT, oxidative resistance, irradiation tolerance, pump rate, and age-related gene expression (*daf-16*, *sod-2*, *sod-3*, *hsp-16.2*, *sir-2.1*, *daf-12*, *jnk-1*) Improved: nuclear localization of DAF-16	[18]
**Anticancer**	Fruit extract	Human breast cancer cells	2, 4, and 6 mg/mL	Antiproliferative activity, EC_50_ of free extract: 4.08 ± 0.09 mg/mL Activated: p53, p21, CDK4, Cyclin E, Bax, and Caspase3 (p53 signaling pathway)	[19]
	Polysaccharide	AsPC-1, BxPC-3, and PANC-1 cells and BALB/cA nu/nu mice	7.45 and 14.9 μM (in vitro) and 0.5 and 40 mg/kg (in vivo)	(in vitro) Decreased: proliferation of pancreatic cancer cells (in vivo) Decreased: tumor sizes, tumor weights, Ki67, CD31, total NF-κB, p-GSK-3β, β-Catenin, p-P38, Bcl-2, caspase-3, and caspase-9 Increased: apoptosis	[20]
	Polysaccharide	AsPC-1, BxPC-3, and PANC-1 cells	4.36 and 8.71 μM	Decreased: proliferation of pancreatic cancer cells, invasion ability, p-AKT, p-GSK-3β, p-FAK, and p-p38	[21]
	Polysaccharide and anthocyanin	LoVo cells and HepG2 cells	Polysaccharide 150, 300, and 500 μg/mL (with anthocyanin 20 μg/mL)	Decreased: proliferation of carcinoma cells Inhibited: replication by G0–G1 arrest Increased: apoptosis	[22]
**Hepatoprotective**	Anthocyanin	Sprague Dawley rats	100 mg/kg	Improved: histological damages Decreased: serum AST, ALT, and LDH levels and *Fas*/*FasL* mRNA expression level (relieved liver cell death)	[13]
	Fruit extract	Male *ApoE*^−/−^ mice	140 mg/kg	Similar: liver morphology, weight, indices of liver/body weight, total bile acid level, serum ALT level, TC, TG, LDL, and HDL-c levels (compared to WD) Decreased: AST levels (compared to NC) and size of fat droplet in liver (compared to WD)	[16]
**Neuroprotective**	Anthocyanin	Neuro-2a cells and Male C57BL/6 mice	10 μM (in vitro) and 50 and 100 mg/kg (in vivo)	(in vitro) Decreased: COX-2, TNF-α, IL-6, IL-1β, and p-NF-κBp65 Increased: cell viability of CML-treated cells Improved: CML-induced apoptosis (in vivo) Decreased: p-NF-κB, TNF-α, IL-1β, IL-6, and caspase-3 (relieved hippocampus neuronal apoptosis) Improved: cognitive impairment (enhanced spatial learning and memory abilities)	[14]
	Anthocyanin	Female Sprague Dawley rats	50, 100, and 200 mg/kg	Decreased: d-gal-Induced neuronal apoptosis, p-JNK, Bax/Bcl-2 ratio, caspase-3, RAGE, BACE-1, Aβ42, GFAP, and Iba-1 Improved: learning, memory impairment, memory ability, and passive avoidance	[17]
	Polysaccharide	Primary cortical neuronal cells in Sprague Dawley rats	0, 5, 10, and 20 μM	Increased: cell viability and expression levels of Nrf2 Inhibited: apoptosis (decreased caspase-3 activity and ratio of bax/bcl-2)	[23]
	Polyphenolic Glycosides	PC12 cells	25, 50, and 100 μM	MAO-B inhibition rates (compounds **1**, **2**, **11**, **16**, and 17/1): IC_50_ value of 60.7 ± 1.7, 22.2 ± 0.8, 79.3 ± 2.4, 68.9 ± 1.5, and 75.5 ± 3.8 μM, respectively Decreased: apoptosis (compounds **1**, **2**, and **6**) Improved: cell viabilities (compounds **1**–**3** and **5**/**6**), morphologic changes (compounds **1** and **2**)	[24]
**Cardioprotective**	Fruit extract	ICR mice	375 and 750 mg/kg	Decreased: CK-MB and LDH activities (amelioration of the myocardial histopathology), fibers necrosis, the number of inflammatory cells, and myocardial tissue (improved myocardial tissue damage)	[25]
**Radiation injury protective**	Fruit extract	Male Kunming mice	2, 4, and 6 g/kg	Decreased: caspase-3, P53, and apoptosis Increased: thymus index, spleen index, and DNA content	[26]
**Immunomodulation**	Fruit extract	Male Kunming mice	2, 4, and 6 g/kg	Increased: thymus index and spleen index	[26]
	Polysaccharide	RAW264.7 cells	25, 50, 100, 200, 500, and 1000 μg/mL	Not significantly changed: cell viability at concentrations of less than 200 μg/mL Increased: NO release (25, 50, 100, and 200 μg/mL; activation of macrophage)	[27]
	Polysaccharide	Female Kunming mice	25, 50, and 100 mg/kg	Increased: thymus index, spleen index, T cell and B cell proliferation, macrophage phagocytosis, serum hemolysin formation, IL-2, IL-6, and TNF-α (immunosuppressed mice)	[28]
	Anthocyanin	Synovial fibroblasts (Isolation from RA patients)	100, 200, and 400 μg/mL	Decreased: SF cell viability, proliferation Not significantly changed: T cells and monocyte/macrophage development	[29]
**Anti-fatigue**	Fruit extract	Kunming mice	0.05, 0.1, 0.2, and 0.5 mg/g	Exercise-induced oxidative stress and inflammation were reduced. Decreased: LDH, TNF-α, IL-1β, IL-2, and IL-6 level Increased: SOD level	[15]
	Polysaccharide	Male BALB/c mice	50, 100, and 200 mg/kg	Decreased: immobility times, BUN, TG, CPK, LDH, and MDA levels Increased: glucose, SOD, and GPx levels LRM helped mobilize TG during exercise and protected microparticles by preventing lipid oxidation by modifying several enzyme activities.	[30]
**Anti-obesity and antidiabetic**	Fruit extract	Male *ApoE*^−/−^ mice	140 mg/kg	Decreased: *Ppar*γ and *Fasn* (compared to NC) and *Srebp1* (compared to WD) Not significantly changed: *Scd*, *Lpl*, and *Lxrα* Increased: *Cpt* (compared to WD)	[16]
	Seed oil	Cell free	0.80, 1.60, 3.20, 6.40, and 12.80 mg/mL	Pancreatic lipase inhibitory activity: IC_50_ value of 12.4 ± 0.1 mg/mL CEase inhibitory activity: IC_50_ value of 2.6 ± 0.1 mg/mL (reversible non-competitive inhibition)	[31]
	Phenylpropanoid derivatives	Cell free	400 μM (Compound **13**: 100, 200, 400, and 800 μM)	Most of the compounds showed weak inhibitory activity. Compound **13** showed an effect similar to that of the positive control (acarbose) and increased the inhibitory effect in a dose-dependent manner.	[32]
**Anti-influenza**	Fruit extract	MDCK cell and influenza virus A/H3N2	15.625, 31.25, 62.5, 125, 250, and 500 μg/mL	Decreased: influenza activity (CC_50_ value was higher than 125 μg/mL) Increased: MDCK cell viability infected with the virus	[33]
**Tyrosinase inhibitory**	Fruit extract and purified anthocyanin	Cell free	0, 0.75, 1.5, 2.25, and 3 mg/mL	Tyrosinase monophenolase inhibitory activity: IC_50_ value of 3.0 ± 0.02 mg/mL (extract) and 1.5 ± 0.058 mg/mL (purified), reversible competitive inhibition, and K_i_ = 39.8 ± 1.4 mg/mL Diphenolase inhibitory activity: 3 mg/mL, 30.8 ± 1.0% (extract) and 3 mg/mL, 42.2 ± 0.77% (purified), reversible uncompetitive inhibition, and K_is_ = 2.4 ± 0.10 mg/mL	[34]

## Data Availability

Data sharing is not applicable to this article.

## Abbreviations

6-OHDA	6-Hydroxydopamine hydrobromide
ABTS	2,2′-Azino-bis(3-ethylbenzothiazoline)-6-sulfonic acid
AGEs	Advanced glycation end products
ALT	Alanine transaminase
AST	Aspartate transaminase
BUN	Blood urea nitrogen
CC_50_	50% Cytotoxicity concentration
CEase	Cholesterol esterase
CK-MB	Creatine kinase-myocardial band
CPK	Creatine phosphokinase
Cy	Cyclophosphamide
d-gal	d-galactose
DPPH	2,2-Diphenyl-1-picrylhydrazyl
FDA	Fluorescein diacetate
GPx	Glutathione peroxidase
H&E	Hematoxylin and eosin
H3N2	Influenza A/Novosibirsk/RII-27192S/2020
IC_50_	50% Inhibitory concentration
ICD	International Classification of Diseases
ICR	Institute of Cancer Research
IL	Interleukin
LDH	Lactate dehydrogenase
LPS	Lipopolysaccharides
LRP3	LRM polysaccharide 3
LRPP5	LRM polysaccharide pectin-5
LRM	Lycium ruthenicum Murray
LRP3-S1	LRM polysaccharide 3-S1
LRP4&AC	LRM polysaccharide 4 and anthocyanin
MAO-B	Monoamine oxidase B
MDA	Malondialdehyde
MDCK	Madin-Darby canine kidney
MTT	3-[4,5-dimethylthiazol-2-yl]-2,5 diphenyl tetrazolium bromide
MTX	Methotrexate
NAFLD	Nonalcoholic fatty liver disease
NC	Normal control
NF-κB	Nuclear factor-κB
NO	Nitric oxide
PI	Propidium iodide
RA	Rheumatoid arthritis
RAGEs	AGEs and their receptors
ROS	Reactive oxygen species
SF	Synovial fibroblasts
SOD	Superoxide dismutase
TG	Triglyceride
TNF-α	Tumor necrosis factor-α
WD	Western diet
